# Brain Networks Connectivity in Mild to Moderate Depression: Resting State fMRI Study with Implications to Nonpharmacological Treatment

**DOI:** 10.1155/2021/8846097

**Published:** 2021-01-15

**Authors:** Dmitry D. Bezmaternykh, Mikhail Ye. Melnikov, Andrey A. Savelov, Lyudmila I. Kozlova, Evgeniy D. Petrovskiy, Kira A. Natarova, Mark B. Shtark

**Affiliations:** ^1^Laboratory of Biofeedback Computer Systems, Institute of Molecular Biology and Biophysics, Federal Research Center of Fundamental and Translational Medicine, Novosibirsk 630060, Russia; ^2^International Tomography Center SB RAS, Novosibirsk 630090, Russia; ^3^International Institute of Psychology and Psychotherapy, Novosibirsk 630007, Russia

## Abstract

Network mechanisms of depression development and especially of improvement from nonpharmacological treatment remain understudied. The current study is aimed at examining brain networks functional connectivity in depressed patients and its dynamics in nonpharmacological treatment. Resting state fMRI data of 21 healthy adults and 51 patients with mild or moderate depression were analyzed with spatial independent component analysis; then, correlations between time series of the components were calculated and compared between-group (study 1). Baseline and repeated-measure data of 14 treated (psychotherapy or fMRI neurofeedback) and 15 untreated depressed participants were similarly analyzed and correlated with changes in depression scores (study 2). Aside from diverse findings, studies 1 and 2 both revealed changes in within-default mode network (DMN) and DMN to executive control network (ECN) connections. Connectivity in one pair, initially lower in depression, decreased in no treatment group and was inversely correlated with Montgomery-Asberg depression score change in treatment group. Weak baseline connectivity in this pair also predicted improvement on Montgomery-Asberg scale in both treatment and no treatment groups. Coupling of another pair, initially stronger in depression, increased in therapy though was unrelated to improvement. The results demonstrate possible role of within-DMN and DMN-ECN functional connectivity in depression treatment and suggest that neural mechanisms of nonpharmacological treatment action may be unrelated to normalization of initially disrupted connectivity.

## 1. Introduction

Depression is a widespread psychiatric disorder associated with a number of different symptoms. The diversity of symptoms implies existence of multiple disruptions of neural circuits in depression, and this may be the reason for diverse findings in studies of brain networks in depression.

A body of research considers default mode network (DMN) as a central one for depression development. Depression is mostly associated with increased functional connectivity (FC) within DMN [1–9] with a few contradicting findings [1, 10–13]. External FC of the DMN is increased to anterior cingulate [1, 14–15], thalamus [14], and pars triangularis [3] and decreased to fusiform gyrus, motor cortices [16], cerebellum, insula [17], thalamus, putamen, and calcarine sulcus [18] with mixed findings for hippocampus [1, 18–19]. On internetwork level, depression is associated with less coupling of DMN and anterior salience network (ASN) [2] and DMN and executive control network (ECN) [1] or more ventral DMN and ECN FC [19].

Within-DMN FC is positively correlated with the number of previous depressive episodes [2] with mixed results for current severity [2, 16]. DMN external connectivity to subgenual anterior cingulate is positively correlated with the duration of current depressive episode [14], while posterior cingulate FC is negatively correlated to Montgomery-Asberg Depression Rating Scale (MADRS) [13] and Hamilton Depression Rating Scale (HAM-D) [18] scores. DMN-ASN [4] and DMN-ECN [8] connectivity is inversely correlated with HAM-D score. However, DMN-ECN links are related to depression severity [20] and rumination levels [19].

Task-positive network disruptions are also frequent in depression. Within-network FC of executive control network (ECN) may be excessive [8, 21–23] or deficient [15, 24] in depression. These findings may be partly explained by increased global ECN intraconnectivity and less FC between its prefrontal and parietal nodes [21]. Network's external connectivity is diminished in depression [25] to cerebellar and primary visual network [26] and to various brain regions [17].

Within-ECN coupling in depressed women is correlated with negative self-directed thoughts [27], and ECN-DMN FC is related to rumination [19]. However, within-ECN, ECN-DMN, and some other external ECN FC are negatively associated with HAM-D score [8, 17].

Anterior salience network (ASN) comprising key cortical emotional areas may be over- [21], under- [2], or normally [1] connected in depression. Increased FC of ASN to left precentral and left angular gyri [28] and to lateral prefrontal areas [22] and effective connectivity of both to and from precuneus [3] is related to depression. Major depression is characterized by weakened ASN FC to medial frontal gyrus and of anterior cingulate to posterior insula, middle temporal gyrus, and cerebellum [28–29].

Within-ASN and ASN-DMN FC are inversely related to number of previous depressive episodes [2] and to HAM-D score [4, 28]. However, ASN FC with prefrontal cortex is associated with subjective depression [22].

Depression may be related to impaired FC of some other networks, e.g., sensorimotor [30–33], ventral and dorsal attention [32–33], language [25], affective [11, 17, 31], visual [23, 33], and audial [23, 33]. Some researchers also proposed spatial brain patterns that they view as networks of depression [34–36] or its certain features like rumination [37] or social emotion disruption [5].

Results of above mentioned studies look rather inconsistent, with many networks demonstrating impairment in few studies only. Evidence for increased or decreased FC of three major networks sounds mostly equivocal. The only solid result is increased within-DMN FC, although even this one is actually a generalization of data from certain brain regions which are different from study to study. The relationships between FC measures and depression severity are unclear for all global networks, including DMN. In some studies, FC findings were unrelated to clinical or behavioral measures [11, 23, 38].

Little is known about modification of network FC in depression following nonpharmacological treatments. Psychotherapy-related results comprise reduced dorsal DMN FC to dorsal anterior cingulate after short course of behavioral activation in subclinical depression [39] and ventral attention network intra- and interconnectivity decrease correlated with improvement on MADRS following the cognitive-behavioral treatment for depression or posttraumatic stress disorder [40]. Brakowski et al. [41] in their review claim that psychotherapy influences predominantly fronto-limbic circuit.

FMRI neurofeedback targets fronto-limbic circuit via either amygdala or prefrontal cortex activity or effective connectivity between these regions. Training of left amygdala upregulation with positive autobiographical memories leads to increase of amygdala FC to number of areas including frontal cortices [42, 43], which is consistent with fronto-limbic FC restoration hypothesis. Right amygdala deactivation training in healthy volunteers also triggered increase in amygdala—lateral prefrontal cortex FC [44]. Last, a proof-of-concept was reported for effective connectivity training aimed at increasing prefrontal influences on amygdala and decreasing of reverse ones in bottom-up direction [45].

Thus, existing data on network FC changes related to psychotherapy and fMRI neurofeedback do not look conclusive. Preliminary data show some fMRI neurofeedback protocols influence FC in fronto-limbic system; however, more research is needed to establish solid brain network correlates of treatment process.

The aim of the current study was to examine network FC differences between healthy volunteers and patients with mild to moderate depression and test their correlations with depression estimates. In parallel, dynamics of network FC were studied in patients who received either no treatment or some nonpharmacological support such as cognitive-behavioral therapy or neurofeedback. Besides, the role of baseline connectivity scores as treatment response predictors, and correlations of neural and clinical changes were estimated. Last, between-group differences were matched to dynamic differences in order to identify networks that differentiate depressed participants from healthy and change along with treatment or spontaneous symptom reduction.

## 2. Materials and Methods

### 2.1. Participants

This study continues our research on network correlates of depression published in [26] with substantially increased sample. The intergroup study involved 21 healthy volunteers received a compensation for a participation and 51 participants featuring mild depression (F32.0), moderate depression (F32.1), or dysthymia (F34.1, single patient) (see Table 1). The dynamic study involved 15 patients with mild or moderate depression scanned twice with 2-3-month interval between the recordings without any treatment received. Eight patients featuring similar conditions received a brief cognitive-behavioral therapy course and 6 patients underwent real-time fMRI neurofeedback course. These groups were also scanned pre- and posttreatment. Subsamples were derived from the major sample of 51 patients.

The sample size for the intergroup study is a tradeoff between preventing false positives and collecting groups of realistic size taking into account MR scanning expenses. Sample size estimation based on expected significance of *p* < 0.05 with any Bonferroni-derived correction (*p* < 0.0009 uncorrected) was not practical from this point of view, so sampling was terminated at the point of expected significance of *p* < 0.01 uncorrected assuming statistical power of 0.8, standard deviation of 0.3 and effect size of 0.8 considered as large so we targeted the most notable effects. Thus, sample size was estimated as *n* = (2.56 + 0.84)^2^ × 2 × 0.3^2^/(0.8 × 0.3)^2^ = 36.125 for one group, which is 72 for two groups that matches our sample: 21 + 51 = 72. Results significant at *p* < 0.05 were initially marked with further elimination of those results that were absent in the dynamics study to partly counter the false positives problem.

The sample of the dynamics study is even more dependent on practical reasons because each patient of the real-time fMRI neurofeedback group received 11 MR scanning sessions (8 training and 3 diagnostic ones). Thus, with more sessions devoted to each patient in our study, our sample size was comparable to or slightly less than ones of the majority similar studies in depression [46–49] excluding few recent large sample ones [42, 50].

All participants were screened to exclude neurological or psychotic level mental disorders, psychotropic medication or drugs severely influencing blood flow, and contraindications to MRI. Depression condition had not to be bipolar, seasonal, or secondary to other disease. IQ > 70 was proven with Raven Progressive Matrices test for all participants, and self-regulation ability was established in treatment groups with 3 sessions of frontal alpha-asymmetry-based electroencephalographic neurofeedback. All the participants signed informed consent prior to inclusion in study. The study protocol was in accordance with Helsinki Declaration and was approved by local ethic board of Institute of Molecular Biology and Biophysics.

### 2.2. fMRI Acquisition

The fMRI study was carried out in the International Tomography Center, Novosibirsk, using a 3 T Ingenia scanner (Philips). Functional T2∗-weighted Ssh echo planar imaging scans were acquired using the following parameters: voxel size 2 × 2 × 5 mm, repetition time/echo time = 2500/35 ms, and fat suppression mode. The reference anatomical image was obtained by the T1W 3D turbo field echo method with a voxel size of 1 × 1 × 1 mm. The instruction for participants was to lie still with eyes closed for 6 minutes.

### 2.3. fMRI Analysis

The first five volumes of each series were discarded to ensure the steady state. The preprocessing of fMRI images was performed with the Matlab (Mathworks, Inc.) and SPM12 (Wellcome Trust Center for Neuroimaging University College London) software. The batch included motion correction, slice timing, normalization of the images to MNI space (resampled at 2 mm3), and smoothing with Gaussian kernel of 8 mm. The default settings were used. Shift to 2 mm or rotation to 2 degrees were considered as excessive head movements. One patient's data were excluded because of head movement and another's due to prominent MR artifacts.

GIFT 3.0.a software was used to perform spatial independent components analysis (ICA). The optimal number of components according to the minimal description length criterion was 20 for volunteers/patients design and 17 for pretreatment/posttreatment design. ICA was performed using Infomax algorithm with the option to reduce the stochasticity namely ICASSO and intensity normalization. The individual dynamics were reconstructed from the group data with the GICA, procedure of reverse reconstruction, for each participant. The extracted components in spatial domain were described by *z*-scores of weight coefficients, which indicated the degree of presence of the component time course in a particular voxel.

The average group activation maps for each component and the coefficients of their spatial correlation with gray, white matter and cerebrospinal fluid masks were constructed. Components correlating with mask of either white matter or cerebrospinal fluid more than with one of grey matter were considered as artifacts and excluded from analysis. After that, the correlations with masks of classical resting state networks were calculated for the remaining components. The primary set of components' maps we used was FMRIB/RIC one https://www.fmrib.ox.ac.uk/datasets/brainmap+rsns/, in cases whereNo comma, no strong match to FMRIB/RIC set was found Stanford maps were also tried http://findlab.stanford.edu/functional_ROIs.html. The composition of the components was determined at the threshold *t* = 2. FNC toolbox (http://mialab.mrn.org/software/fnc) was used to calculate temporal correlations between the dynamics of the selected components.

With the Lag-Shift algorithm, the coefficients and the lag times for each pair of networks were computed. The time shift was selected to maximize an absolute value of correlation coefficient. Intergroup differences were estimated with the Student's *t* test for independent samples, the dynamics of the networks intercorrelations from the first to the second recording—with the *t* test for paired samples.

For study 1, in each participant, the Pearson correlation coefficient between time series of the components and 6 rigid body head motion parameters were calculated. Additional tests were performed excluding all pairs involving components with correlation coefficient with any motion parameter above margin in certain participant. *R* > 0.5, *r* > 0.4, and all *p* < 0.05 were tested, and *r* > 0.4 was empirically chosen as a tradeoff between reducing maximal allowed correlation and preserving the majority of data units (85.8%). Thus, *t* test was repeated with exclusion of components correlating with motions to degree of 0.4 or higher, so results significant at first test with all data included and nonsignificant at second test with some data excluded were considered as motion-related false positives. For dynamic comparisons, no such additions were implemented for small samples. Interactions between functional connectivity and depression scales were measured with Spearman correlation implemented in IBM SPSS 21.0 software.

### 2.4. Clinical and Psychological Measures

Russian versions of Beck Depression Inventory (BDI) and Zung Self Rating Depression Scale (ZSRDS) were introduced to participants. Ones who were observed in dynamics filled the forms twice, at the start and finish point. Treatment groups also were assessed with Montgomery-Asberg depression rating scale (MADRS) by an experienced psychiatrist during the interview in the beginning and end of treatment course. Some participants did not show up for the post-course assessment which led to some missing data points.

### 2.5. Treatments

Cognitive behavioral therapy sessions took place in a special room in the Institution of Molecular Biology and Biophysics. A psychiatrist and a clinical psychologist together led treatment groups of five patients at a time. Course covered such topics as ABC and ABCDE models, automatic thoughts detection, links between automatic thoughts and emotions, cognitive distortions, thoughts modification techniques, positive reappraisal, and assertiveness. So, course involved some training and educational aspects. Individual treatment led by one of the specialists comprised more personalized and symptom-focused intervention, problems detection, work with priorities, belief-emotion links, automatic thoughts, and cognitive distortions detection and modification, practicing in ABC model. It also dealt with behavioral and motivational difficulties and included home assignments after each session. In total, each participant of this group received 8 individual and 8 group sessions.

Neurofeedback was performed at the International Tomographic Center with the facility discussed in 2.2.1 and aimed at improving the participants' ability to regulate left medial prefrontal cortex which is supposed to be involved in positive emotions regulation through connections to amygdala. The total scanning time was approximately 30 minutes for each session. Five minutes were spent on the placement of participant into scanner and acquisition of reference images, and 25 minutes were devoted to neurofeedback per se. On even sessions, participants spent 10 minutes of neurofeedback time for a transfer run in which they received no feedback and had to rely on their established strategies of signal regulation. In total, each participant of this group received 8 individual sessions.

## 3. Results

**Figure 1:**
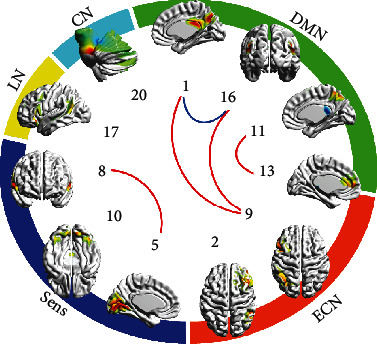
Results of depressed vs. controls comparison. IC numbers match ones of Tables 2 and 3. IC spatial distribution on the most representative cerebral (cerebellar for #20) surface is given. These surface maps were prepared using BrainNet Viewer software. ICs are grouped by relation to functional specialization to DMN, ECN, sensor, language, and cerebellar. Blue lines show pairs with more connectivity in controls, red lines show pairs with more connectivity in depressed patients (*p* < 0.05 uncorrected).

In study 1, 11 of 20 components suited criterion of grey matter prevalence (see Table 2), which led to 55 pairs. Seven pairs demonstrated intergroup differences significant at *p* < 0.05 (see Table 3). After exclusion of motion-correlated data, five of them still featured significant differences (see Table 3), assuming these results were unrelated to motion (Figure 1).

FC in two pairs was demonstrated to be slightly positively correlated with ZSRDS scores in combined group of healthy and depressed participants (see Table 4). No significant results were found in separate groups.

**Figure 2:**
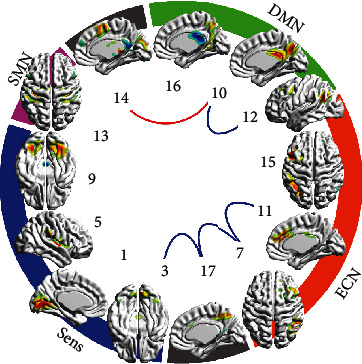
Results of baseline vs. repeated scanning comparison in no treatment group. IC numbers match ones of Tables 5 and 6. IC spatial distribution on the most representative cerebral surface is given. These surface maps were prepared using BrainNet Viewer software. ICs are grouped by relation to functional specialization to DMN, ECN, sensor, sensorimotor, and not matching classical networks. Blue lines show pairs with more connectivity at baseline—decline in time, red lines show pairs with more connectivity at the second measurement—growth in time (*p* < 0.05 uncorrected).

**Figure 3:**
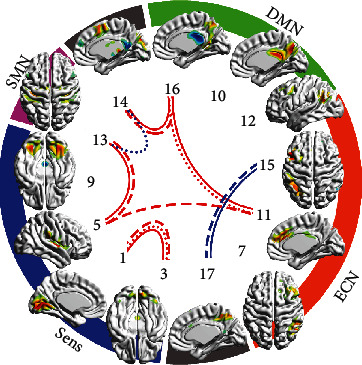
Results of baseline vs. posttreatment comparison in treatment groups. IC numbers match ones of Tables 5 and 6. IC spatial distribution on the most representative cerebral surface is given. These surface maps were prepared using BrainNet Viewer software. ICs are grouped by relation to functional specialization to DMN, ECN, sensor, sensorimotor, and not matching classical networks. Blue lines show pairs with more connectivity at baseline—decline in time, red lines show pairs with more connectivity posttreatment—growth in time (*p* < 0.05 uncorrected). Solid lines indicate both groups together, dashed lines—CBT, dotted lines—NFB.

In study 2, 13 of 17 components were considered as grey matter ones (see Table 5), so 78 pairs were tested. Differences in a few pairs were found in patients who did not receive the treatment (see Table 6, Figure 2). FC in some pairs changed after the psychotherapy course or after neurofeedback course (see Table 6, Figure 3). FC in a few pairs changed while considering both treatment groups as one sample (see Table 6, Figure 3).

Among pairs mentioned in dynamic comparisons, 1-3 and 5-11 changes were correlated with ZSRDS scores' dynamics in no treatment group positively and negatively, respectively (see Table 7). Psychotherapy group featured no such correlations, while in neurofeedback group, 1-3 and 5-13 FC changes were positively related to ZSRDS score dynamics, and 3-17 and 7-17 were negatively correlated with MADRS score changes (see Tables 8 and 9). 1-3 changes were associated positively with BDI and ZSRDS score dynamics, while 5-11 to ZSRDS only; 10-12 changes were related inversely to MADRS scores changes in combined treatment groups (see Table 10).

When depression scores' changes were correlated with baseline connectivity scores of the pairs that featured a significant dynamic changes in FC (which means identifying network predictors of clinical improvement), the following results were demonstrated (Tables 7–10). In no treatment group, ZSRDS score change was positively correlated with 5-11 and 10-12 pairs baseline FC, while BDI score increase was negatively related to initial FC in 3-17, 7-11, and 14-16 pairs. In combined treatment group, 7-17 coupling was inversely related to MADRS score change and 10-12 was positively correlated to estimates of MADRS and ZSRDS. In psychotherapy group, 3-17 initial FC was negatively linked to ZSRDS score change. In neurofeedback group, 1-3 start FC was negatively associated with ZSRDS score dynamics, while MADRS change was inversely correlated with 5-11 baseline FC and positively with 10-12 FC.

**Figure 4:**
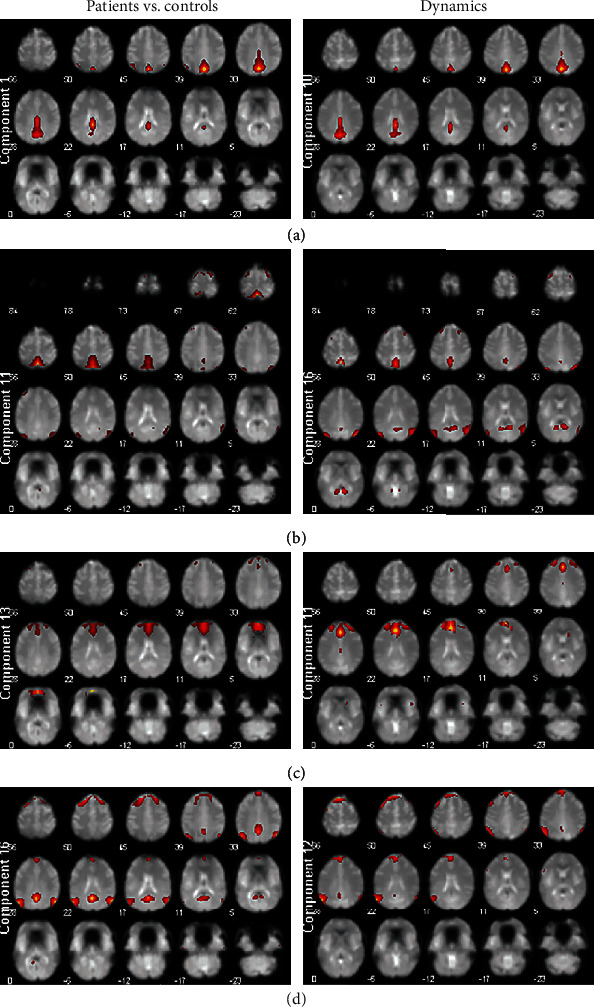
Slice-based maps of matched components demonstrating significant differences in both studies. In each panel, left map suits to depressed vs. controls study (IC number from the Table 2), and right one suits to baseline vs. repeated measure study (IC number from the Table 5). Slice rows with no or negligible activity are omitted. (a) IC1 from study 1 and IC10 from study 2; (b) IC11 from study 1 and IC16 from study 2; (c) IC13 from study 1 and IC11 from study 2; (d) IC16 from study 1 and IC12 from study 2.

Correlations of component masks of bigger and smaller samples were computed (see Table 11). Only two pairs of components were identified both in first and second parts of study: 1-16 (10-12 in dynamics test) and 11-13 (11-16 in dynamics test) (Figure 4).

## 4. Discussion

### 4.1. Study 1: Intergroup Results

The first result is decreased within-DMN FC in depressed group, with one component of the pair suiting classical DMN topography including portions of precuneus, posterior cingulate, bilateral temporo-parietal junction, and medial prefrontal cortex, and another is located mostly in posterior cingulate and precuneus. As mentioned in the introduction section, most studies in the field highlighted within-DMN overconnectivity among features of depression. This is the most reliable functional connectivity marker of depression across studies. Moreover, depression-related DMN nodes FC show reliability of 0.5-0.76 [19]. Note that increased FC of components 11 and 13 in our study aside from DMN–ECN connectivity represents coupling of precuneus and portion of medial prefrontal cortex. However, some studies indicating underconnectivity of posterior cingulate within the DMN also exist. According to [10, 13], patients with major depression lack connection of posterior cingulate with prefrontal cortex and temporo-parietal area. Sad mood induction in depression leads to posterior cingulate uncoupling from the prefrontal cortex and precuneus [51]. Some antidepressant medications increase FC of posterior cingulate to medial prefrontal area [52], and psychotherapy increases it to precuneus [38], which may suggest that low strength of these connections is related to depression.

So decreased FC of these DMN subsystems is not in line with majority of the studies, yet has some limited support from the previous research. DMN is known for its role in processing of internal states including both psychological and physical, self- and close others-related information, and for some kinds of social cognition like theory of mind [53]. Disruption of connections between the nodes of the system may reflect difficulties in some social skills requiring applying other's perspectives (empathy, emotional intelligence, theory of mind) or in relationships with close others which are frequent in depression.

Three results suggest that DMN subnetworks are linked to task-positive networks to a larger degree in depression, namely, posterior cingulate/precuneus and multinode DMN component are overconnected with left fronto-parietal areas, while superior precuneus is hypersynchronized with bilateral frontal component related mostly to ECN. This implicates disruption of normal relationships between three key networks of triple network model [54], namely, DMN, ECN, and ASN assuming DMN is anticorrelated with two others. Global increase of DMN–ECN FC is not a typical finding in depression (see results directly contradicting ours in [8] and indirectly in [38, 52]); however, it is possibly related to some depression-specific cognitive processes [19]. So the difference between ours and previous results may be caused by relatively mild and supposedly more “psychogenic” conditions in our case lacking some neural markers typical for more serious conditions while sharing cognitive features of depression such as rumination and cognitive control deficits.

Note that DMN–left frontoparietal network is the pair discriminating between healthy and depressed people to the highest degree and the only one showing significant correlation with depression assessment score when groups are combined. This may show the importance of laterality and be related to frontal asymmetry described in models by Davidson and Heller. According to these models, left prefrontal activity is related to positive emotions and to approach motivation, while right corresponds to negative emotions and withdrawal motivation (see [55] for a review). Relatively active right prefrontal area and idling left prefrontal cortex together may be a neurophysiological signature of depression. From this point, increased coupling of the network containing left dorsolateral prefrontal area with DMN may indicate its passivity resulting in less approach motivation and less positive mood which is one of the most important depression-related signs.

The last result is an overconnectivity of medial visual cortex with audial cortex in depression. Sensor systems are mentioned in few depression studies to date. FC between visual and audial networks is disrupted in depression and may be used as a marker of a disorder [33]. Depressed patients are known to spend less time in a state of strong connectivity between auditory and visual networks [8], which is in contrast with our results. More research on the role of sensor networks in depression should be conducted to interpret it accurately.

### 4.2. Study 2: Treatment-Related Results

Functional connectivity of fronto-parietal networks and key task-positive systems, are of great interest. Right fronto-parietal network diminishes its coupling with other ECN areas and also with superior parietal region in no treatment group. Left fronto-parietal network also decreases FC with superior parietal component. Common tendency that corresponds to normal global connectivity pattern may be an isolation of both fronto-parietal networks from some task-negative regions. Moreover, right fronto-parietal areas become less connected with ECN frontal areas, including prefrontal cortex and anterior cingulate. This may be interpreted in terms of functional frontal asymmetry mentioned in a previous subsection. Isolation of right frontal cortex especially from task positive networks may contribute to mood improvement for reduction of negative emotions. No strong prevalence of data on under- or overconnectivity within-ECN in depression or on increasing or decreasing of ECN areas FC with task-negative networks exist in literature (see Introduction); thus, diminished coupling revealed in our study is partly supported by previous research.

In treatment groups, DMN FC increases to supplementary motor area and to frontal ECN, with coupling to SMN driven by CBT and to ECN by NFB. Baseline DMN–SMN synchronicity is positively related to subsequent improvement in no treatment group. Some data suggest that low DMN FC with motor areas is typical for depression [16, 32], so its increase may be treated as probably associated with an improvement. Indirect data indicate positive association between DMN–ECN coupling and some features of depression [19–20], though direct evidence [1, 8] along with our intergroup comparison results demonstrates decreased DMN–ECN FC in depression. Increase of this connection of naturally un- or anticorrelated networks may be even more dysfunctional in terms of global networks interrelations, yet no link to clinical measures is present in our study.

FC within visual system (between occipital pole and medial visual cortex) grows during any treatment course (marginally significantly in CBT group). However, this FC change is positively related to depression scores change which means inverse links to improvement both in treatment and in no treatment conditions. In neurofeedback group, baseline coupling of this pair is negatively correlated to change on Zung scale during treatment, so strength of connectivity in this pair is a predictor of success. These results may be interpreted as a presence of a neural mechanism involving visual systems of occipital cortex which is frequently activated during the treatment of depression yet decreases its effectiveness. The role of FC in this pair as a predictor may be that initial high coupling does not leave enough room for increase in this connectivity score. Depressed patients have less FC [23] and spend less time in a state of increased connectivity of networks within visual system [8, 9], so increase in this connection may be related to improvement. Note also that in no treatment group, FC of occipital pole with a superior parietal component increased which may indicate disruption of the dorsal stream of visual processing or isolation of visual system from the DMN. However, medial visual network does not share this pattern, so interpretation in terms of whole visual system would sound premature. Occipital pole–superior parietal FC at baseline is related to treatment success of CBT and to degree of spontaneous improvement, and its dynamics is also correlated with improvement due to NFB. Thus, in contrast to previously discussed connections increasing in time yet inversely related to improvement, coupling in this pair decreases over time yet directly related to improvement.

Strength of a few connections of auditory system, namely, to SMN and ECN, increases in CBT. This result is supported by data on weak ECN coupling with temporal regions in clinical [8, 17] and subclinical [56] depression, yet some contradicting results also exist [3]. SMN–auditory networks FC also may be in deficit in depression [8]. Despite of not being directly associated with emotions, SMN–auditory network connections may be valuable features of depression [33]. From the dynamic point of view, changes in auditory system FC to ECN are associated with more improvement in no treatment group and less improvement in a combined treatment group. Baseline score of this pair is a negative predictor for no treatment group and positive in neurofeedback group. Auditory–SMN FC is also negatively related to outcome in NFB group.

Last, a decrease of FC within sensorimotor system is detected after neurofeedback course, namely, between components representing paracentral lobule and supplementary motor cortex, and not correlated with clinical estimates. Previous research mentioned within-SMN connectivity among other markers of depression [30, 33] mostly indicating its deficit [8]. Thus, while neurophysiological mechanism of this link is uncertain, integration of different sensor, sensorimotor, and, possibly, other task-positive systems may correspond to some clinical change in depression depending on kind of intervention.

While observing FC changes in a no treatment group and in treatment groups as a whole, one can see that dynamics in a no treatment group are mostly decreasing of FC, and in treatment groups, they are related primarily to the increasing of FC. Certain IC pairs are not crossing over between these conditions. So distinct neurophysiological mechanisms may be suggested in spontaneous depression improvement and in treatment-related one, involving different brain networks and antagonistic mechanisms in terms of FC. Yet this idea requires a thorough investigation with larger samples, to our knowledge, it is the first conceptual neuroscientific approach to specificity of spontaneous improvement in depression.

### 4.3. Integration of the Data from Two Studies

According to the correlation matrix, most of the ICs in study 1 are strongly correlated with sole component in the study 2. Only few ICs show ambiguous correlations or do not show them at all. Our aim was to identify network connections disrupted in depression (study 1) and changed in the treatment course (study 2), so we performed a search through our results (see Table 11, Figure 4).

First, 1-16 pair representing within-DMN connectivity of posterior cingulate/precuneus region with initially decreased coupling in depression corresponds mostly to 10-12 pair of the dynamic study. It shows links between posterior cingulate/precuneus node and hybrid network including left frontal cortex like fronto-parietal network and left temporo-parietal area with a portion of posterior cingulate like DMN. Visual inspection shows high equivalence of posterior cingulate/precuneus components in both studies, while the whole-DMN component of study 1 matches IC with no bilateral temporo-parietal pattern and less prominent midline parietal activation. Nevertheless, these pairs may be considered as relatively equivalent. We should remind that connectivity in the 10-12 pair even more decreases in no treatment condition, and its baseline estimate is negatively correlated with treatment success in no treatment group, combined treatment group, and NFB group. Moreover, its change is positively related to treatment effectiveness in a combined treatment group. Thus, this result, if not treated as a false positive, reveals complicated dynamics of brain functional changes in depression and in recovery from depression (most people from no treatment group spontaneously improved on their depression estimates). Thus, FC of DMN may be treated not as a pathological sign, but as a kind of protective or compensatory mechanism activated in some cases (multiple articles showing overconnectivity of DMN in depression) and not activated in others (other studies including ours). Interestingly, it generally fades away with time, while patients preserving this connection relatively strong seem to benefit more from nonpharmacological treatment.

Second, FC in 11-13 pair in study 1 (precuneus with prefrontal cortex suiting partly within-DMN and partly DMN–ECN synchronicity) augmented in depression group matches 11-16 pair of study 2. This pair defines coupling between an IC of precuneus and temporo-parietal junction (DMN) with another one covering a large portion of prefrontal cortex, mostly medial, with anterior cingulate and associated primarily with ECN. Frontal systems in studies 1 and 2 are topographically similar, while DMN components feature some differences not discarding their relation to DMN. FC in this pair grows posttreatment in combined group and in NFB group; however, neither its baseline score, nor its changes predict clinical outcomes. So in contrast to previous pair coupling initially lower in depression and decreasing over time, this pair FC is higher in depression and even increases after treatment.

These data show that common approach of searching for fMRI biomarkers of depression and considering their changes to normal values as a success indicator may be inappropriate in some cases, for in our study network connectivity patterns changed in “more pathological” direction yet symptoms improved over time, so both no treatment and treatment condition were not deleterious. Abnormal values of FC may be associated, aside from disorder symptoms, with some compensation processes or condition- and symptom-unrelated changes. They also may be either state- or trait-related, and modification of trait-related functional brain changes seems questionable.

Collected data support crucial role of within-DMN and DMN-ECN FC in depression and demonstrate need for a further study of the relationships between these networks coupling and development and treatment of depression for using a relatively big sample of depressed patients we found decrease of within-DMN FC that contradicts typical findings in the field. Interrelations between depression severity, recurrence and aetiology, and network organization should be examined thoroughly for these data were collected in a sample of participants with relatively mild and predominantly psychogenic depression. In treatment effects on brain connectivity, more research of nonpharmacological treatments is needed.

### 4.4. Clinical Implications

The largest portion of the discussion above was devoted to network neuroscience features of depression and more to fundamental than to applied science. So we summarize practical implications of the article in a separate subsection.

First, FC biomarkers of depression do not look solid and universal because our study revealed some results contrary to the mainstream of the existing body of research. Inconsistencies may be related to sample differences (depression severity, presence or absence of some certain symptoms, racial/ethnic sample composition, and other demographics) or details in acquisition and analysis of fMRI data. Thus, resting state FC biomarkers of depression are premature for usage in diagnostics and evaluation of treatment success in depression.

Second, across potential FC features of depression itself and of improvement from depression, our results suggest that left frontoparietal network coupling to DMN and right frontoparietal network coupling to ECN are of use. These findings support usage of approaches targeting prefrontal cortices in mild depression with respect to activity lateralization, such as EEG and fMRI neurofeedback, transcranial direct current stimulation, and transcranial magnetic stimulation of prefrontal cortex.

Third, according to our data, DMN hyperconnectivity revealed in the majority of studies of depression should be treated as a target of neuromodulation with great caution for our study shows its presence in depression is ц optional, and correlational analysis shows it may be related to spontaneous compensatory processes, not to disorder itself.

## 5. Limitations

The key limitation of the study is an approach to results extraction. Corrections for multiple comparisons were not implemented, and none of the results mentioned would reach corrected significance level. Instead, we considered valuable effects present both in study 1 and study 2 that addresses possibility of false positives from logical point of view and not from statistical one. The second limitation is a small size of dynamic groups which could be a source of false positives for absence of multiple comparison corrections. Third limitation is an incomplete match of components of study 1 and study 2 that may rise the question of equivalence of the corresponding ICs.

## 6. Conclusions

Though no intergroup results reached a corrected significance level, the most prominent differences were increased functional connectivity between left frontoparietal network and subsystems of the DMN. Intergroup differences reflected also in dynamic comparisons were (1) decreased within-DMN FC even more diminished over time and negatively related to treatment outcome and (2) increased DMN–ECN FC augmented after neurofeedback treatment. These results contribute to model of frontal emotional asymmetry in depression, demonstrate deficient DMN connectivity in depression in contrast to majority of the studies published to date, and show need of further investigation of interrelations of three global networks in depression.

## Figures and Tables

**Table 1:** Demographic and clinical characteristics of the groups involved in the study.

Group	Sex	Age, Mean ± SD	IQ, Mean ± SD	MADRS, Mean ± SD	BDI, Mean ± SD	ZSRDS, Mean ± SD
Healthy controls, *N* = 21 (HC)	6 M, 15 F	33.8 ± 8.5	106.0 ± 16.1	—	4.6 ± 4.5	32.1 ± 5.9
Total depressed, *N* = 51 (DEP-51)	13 M, 38 F	33.1 ± 9.5	103.7 ± 14.6	26.7 ± 4.4	20.7 ± 10.0	46.4 ± 7.0
No treatment, *N* = 15 (DEP-NT)	5 M, 10 F	35.2 ± 9.4	100.7 ± 15.2	—	18.4 ± 11.2	44.6 ± 8.3
Treatment, *N* = 14 (DEP-TR)	3 M, 11 F	29.8 ± 8.7	104.7 ± 12.7	28.4 ± 2.4	25.9 ± 10.0	50.9 ± 5.1
Psychotherapy, *N* = 8 (DEP-CBT)	3 M, 5 F	29.1 ± 8.2	109.8 ± 11.1	28.4 ± 2.9	24.1 ± 8.9	48.3 ± 4.6
Neurofeedback, *N* = 6 (DEP-NFB)	6 F	30.7 ± 10.2	96.6 ± 11.5	28.4 ± 1.9	28.3 ± 11.7	55.2 ± 2.3

*N*: sample size; M: males; F: females; SD: standard deviation; MADRS: Montgomery-Asberg depression rating scale; BDI: Beck depression inventory; ZSRDS: Zung self-rating depression scale. DEP-NT, DEP-TR, DEP-CBT, and DEP-NFB groups are parts of DEP-51 sample.

**Table 2:** Independent component analysis results for depressed vs. controls comparison.

IC no.	GM	WM	CSF	Accepted	Best match
1	0.07	-0.05	0.00	Yes	Default mode network (*r* = 0.69)
2	0.11	-0.09	-0.03	Yes	Right frontoparietal network (*r* = 0.69)
3	0.15	-0.19	0.17	No	
4	0.06	-0.16	0.29	No	
5	0.11	-0.07	-0.03	Yes	Medial visual network (*r* = 0.82)
6	0.02	-0.02	0.01	No	
7	0.00	-0.05	0.12	No	
8	0.20	-0.19	0.02	Yes	Audial network (*r* = 0.62)
9	0.08	-0.07	-0.02	Yes	Left frontoparietal network (*r* = 0.76)
10	0.23	-0.20	-0.03	Yes	Lateral visual network (*r* = 0.46), occipital pole (*r* = 0.40)
11	0.11	-0.11	0.05	Yes	–/Stanford ventral DMN (*r* = 0.48)
12	0.14	-0.17	0.12	No	
13	0.01	0.01	-0.02	Yes	Executive control network (*r* = 0.62)/Stanford dorsal DMN (*r* = 0.43)
14	0.26	-0.25	0.11	No^∗^	
15	-0.44	0.36	0.05	No	
16	0.21	-0.21	0.06	Yes	Default mode network (*r* = 0.57)
17	0.15	-0.19	0.07	Yes	–/Stanford language network (*r* = 0.43)
18	-0.06	0.10	-0.08	No	
19	0.09	-0.12	0.06	No	
20	0.17	-0.17	0.09	Yes	Cerebellum network (*r* = 0.37)

IC: independent component; GM: gray matter; WM: white matter; CSF: cerebro-spinal fluid; *r*: correlation coefficient; ^∗^excluded for an artifact localization based on visual examination.

**Table 3:** Results of depressed vs. controls comparison in all cases (left part) and in cases correlated with 6 solid body motion parameters less than *r* = 0.4 on individual level (right part).

Pair	All cases				*r* < 0.4 with motion parameters			
	HC, Mean ± SD	DEP-51, Mean ± SD	*t*	*p*	HC, Mean ± SD	DEP-51, Mean ± SD	*t*	*p*
1-9 (DMN-LFr)	–0.09 ± 0.27	0.14 ± 0.32	–2.93	0.005	–0.11 ± 0.27	0.13 ± 0.33	–2.88	0.005
1-16 (DMN-DMN)	0.52 ± 0.19	0.35 ± 0.38	2.59	0.012	0.54 ± 0.19	0.38 ± 0.34	2.34	0.023
2-20 (RFr-Cer)	0.04 ± 0.32	0.21 ± 0.30	–2.13	0.037	0.02 ± 0.33	0.19 ± 0.31	–1.99	0.051 (n/s)
5-8 (mVis-AN)	0.15 ± 0.36	0.37 ± 0.38	–2.32	0.023	0.15 ± 0.36	0.39 ± 0.36	–2.50	0.015
5-17 (mVis-LN)	0.13 ± 0.29	–0.02 ± 0.35	1.89	0.066 (n/s)	0.15 ± 0.28	–0.10 ± 0.32	2.96	0.005
9-16 (LFr-DMN)	–0.12 ± 0.21	0.07 ± 0.29	–2.97	0.004	–0.13 ± 0.21	0.08 ± 0.29	–3.31	0.002
11-13 (DMN-ECN)	0.03 ± 0.37	0.23 ± 0.30	–2.34	0.022	0.02 ± 0.38	0.23 ± 0.31	–2.27	0.027
11-17 (DMN-LN)	0.04 ± 0.30	0.18 ± 0.34	–2.13	0.037	–0.16 ± 0.30	0.01 ± 0.32	–1.97	0.055 (n/s)

SD: standard deviation; DMN: default mode network; LFr: left fronto-parietal network; RFr: right fronto-parietal network; Cer: cerebellar network; mVis: medial visual network; AN: audial network; LN: language network; ECN: executive control network; *t*: *t* test value; *p*: 2-tailed significance level; n/s: nonsignificant.

**Table 4:** Correlations of connectivity in IC pairs and depression scores.

	Both groups—ZSRDS	
Pair	*r*	*p*
1-9 (DMN-LFr)	0.273	0.026
1-16 (DMN-DMN)	–0.129	n/s
5-8 (mVis-AN)	0.116	n/s
9-16 (LFr-DMN)	0.296	0.016
11-13 (DMN-ECN)	0.196	n/s

DEP-51 and HC groups taken together; analysis on separate groups led to no correlations significant at *p* < 0.05. DMN: default mode network; LFr: left fronto-parietal network; mVis: medial visual network; AN: audial network; ECN: executive control network; *r*: correlation coefficient; *p*: 2-tailed significance level; n/s: nonsignificant.

**Table 5:** Independent component analysis results for dynamics study.

IC no.	GM	WM	CSF	Accepted	Best match
1	0.20	0.09	0.08	Yes	Medial visual network (*r* = 0.78)
2	0.14	0.03	0.15	No	
3	0.21	0.05	0.17	Yes	Occipital pole (*r* = 0.36)
4	0.19	0.01	0.25	No	
5	0.27	0.08	0.13	Yes	Audial network (*r* = 0.69)
6	0.24	0.17	0.21	No	
7	0.21	0.12	0.08	Yes	Right frontoparietal network (*r* = 0.71)
8	0.16	0.03	0.31	No	
9	0.28	0.13	0.07	Yes	Lateral visual network (*r* = 0.59)
10	0.18	0.10	0.08	Yes	Default mode network (*r* = 0.75)
11	0.23	0.17	0.11	Yes	Executive control network (*r* = 0.64)
12	0.22	0.11	0.11	Yes	Default mode network (*r* = 0.31), left frontoparietal network (*r* = 0.29)
13	0.22	0.20	0.14	Yes	Sensorimotor network (*r* = 0.66)
14	0.26	0.17	0.22	Yes	—
15	0.22	0.17	0.10	Yes	Left frontoparietal network (*r* = 0.75)
16	0.24	0.15	0.17	Yes	Default mode network (*r* = 0.39)
17	0.25	0.17	0.11	Yes	—

IC: independent component; GM: gray matter; WM: white matter; CSF: cerebro-spinal fluid; *r*: correlation coefficient.

**Table 6:** Results of baseline vs. repeated measure comparison in no-treatment group (*N* = 15), combined treatment group (*N* = 14), CBT group (*N* = 8), and NFB group (*N* = 6).

Pair	Pre, Mean ± SD	Post, Mean ± SD	*t*	*p*
*No treatment group*				
3–17 (OccP–?)	0.16 ± 0.30	–0.01 ± 0.36	2.21	0.044
7–11 (RFr–ECN)	0.31 ± 0.12	–0.02 ± 0.33	3.75	0.002
7–17 (RFR–?)	0.31 ± 0.16	0.09 ± 0.26	2.58	0.022
10–12 (DMN–DMN/LFr)	0.45 ± 0.15	0.31 ± 0.18	2.21	0.044
*10–14 (DMN–?)*	–0.49 ± 0.17	–0.34 ± 0.22	–2.34	0.035
*Combined treatment group*				
1–3 (mVis–OccP)	0.17 ± 0.40	0.50 ± 0.22	–3.71	0.003
5–13 (AN–SMN)	0.43 ± 0.33	0.55 ± 0.27	–2.61	0.022
11–16 (ECN–DMN)	0.14 ± 0.36	0.37 ± 0.22	–2.44	0.030
14–16 (?–DMN)	–0.01 ± 0.34	0.19 ± 0.25	–2.48	0.028
15–17 (LFr–?)	0.25 ± 0.3	–0.01 ± 0.35	2.21	0.046
*Cognitive behavioral therapy group*				
1–3^∗^ (mVis–OccP)	0.30 ± 0.38	0.53 ± 0.28	–2.35	0.051 (n/s)
5–11 (AN–ECN)	0.36 ± 0.32	0.54 ± 0.24	–3.53	0.010
5–13 (AN–SMN)	0.45 ± 0.30	0.61 ± 0.15	–2.39	0.049
14–16 (?–DMN)	–0.07 ± 0.39	0.22 ± 0.26	–2.85	0.026
15–17 (LFr–?)	0.28 ± 0.35	–0.13 ± 0.38	2.50	0.040
*fMRI neurofeedback group*				
1–3 (mVis–OccP)	0.01 ± 0.39	0.47 ± 0.14	–3.00	0.030
11–16 (ECN–DMN)	–0.10 ± 0.17	0.28 ± 0.26	–3.35	0.020
13–14 (SMN–?)	0.30 ± 0.26	–0.04 ± 0.42	3.26	0.022

SD: standard deviation; DMN: default mode network; LFr: left fronto-parietal network; RFr: right fronto-parietal network; mVis: medial visual network; AN: audial network; ECN: executive control network; SMN: sensorimotor network; OccP: occipital pole network; ?: IC does not match any classical network; *t*: *t* test value; *p*: 2-tailed significance level; n/s: nonsignificant. ^∗^marginally significant result is given for it corresponds to significant results in NFB group and in combined treatment group.

**Table 7:** Correlations of baseline scores and pre-post changes in connectivity with pre-post changes in clinical/psychological variables in no-treatment group (*N* = 14).

IC pair	BDI		ZSRDS	
	Baseline	Change	Baseline	Change
1–3 (mVis–OccP)	0.056	0.112	–0.354	0.625^∗^
3–17^a^ (OccP–?)	–0.637^∗^	–0.105	–0.38	–0.323
5–11 (AN–ECN)	0.363	–0.32	0.701^∗∗^	–0.674^∗∗^
7–11^a^ (RFr–ECN)	–0.677^∗∗^	0.479	–0.372	0.347
10–12^a^ (DMN–DMN/LFr)	0.456	–0.02	0.63^∗^	–0.343
14–16 (?–DMN)	–0.598^∗^	0.327	–0.223	0.298

DMN: default mode network; LFr: left fronto-parietal network; RFr: right fronto-parietal network; mVis: medial visual network; AN: audial network; ECN: executive control network; OccP: occipital pole network; ?: IC does not match any classical network; BDI: Beck depression inventory; ZSRDS: Zung self-rating depression scale; ^∗^*p* < 0.05; ^∗∗^*p* < 0.01; ^a^pre-post difference in connectivity of this pair is significant for this condition.

**Table 8:** Correlation of baseline scores and pre-post changes in connectivity with pre-post changes in clinical/psychological variables in CBT group (*N* = 5).

IC pair	ZSRDS	
	Baseline	Change
3–17 (OccP–?)	–0.949^∗^	–0.158

OccP: occipital pole network; ?: IC does not match any classical network; ZSRDS: Zung self-rating depression scale; ^∗^*p* < 0.05.

**Table 9:** Correlation of baseline scores and pre-post changes in connectivity with pre-post changes in clinical/psychological variables in NFB group (*N* = 4 − 6).

IC pair	MADRS		ZSRDS	
	Baseline	Change	Baseline	Change
1–3^a^ (mVis– OccP)	0	0	–0.9^∗^	1^∗∗^
3–17 (OccP–?)	0.8	–1^∗∗^	0.7	–0.4
5–11 (AN–ECN)	–1^∗∗^	0.8	–0.3	0.5
5–13 (AN–SMN)	–0.4	0.8	–0.5	0.9^∗^
7–17 (RFr–?)	0.2	–1^∗∗^	–0.2	–0.7
10–12 (DMN–DMN/LFr)	1^∗∗^	–0.8	0.8	–0.6

DMN: default mode network; LFr: left fronto-parietal network; RFr: right fronto-parietal network; mVis: medial visual network; AN: audial network; ECN: executive control network; SMN: sensorimotor network; OccP: occipital pole network; ?: IC does not match any classical network; MADRS: Montgomery-Asberg depression rating scale; ZSRDS: Zung self-rating depression scale; ^∗^*p* < 0.05; ^∗∗^*p* < 0.01; ^a^pre-post difference in connectivity of this pair is significant for this condition.

**Table 10:** Correlation of baseline scores and pre-post changes in connectivity with pre-post changes in clinical/psychological variables in a combined treatment group (*N* = 10 − 11).

IC pair	MADRS		BDI		ZSRDS	
	Baseline	Change	Baseline	Change	Baseline	Change
1–3^a^ (mVis– OccP)	–0.156	0.361	–0.489	0.615^∗^	–0.627	0.774^∗∗^
5–11 (AN–ECN)	0.009	0.202	–0.265	0.469	–0.492	0.64^∗^
7–17^a^ (RFr–?)	–0.672^∗^	0.41	0.043	–0.123	0.064	–0.299
10–12 (DMN–DMN/LFr)	0.716^∗^	–0.734^∗^	0.51	–0.369	0.646^∗^	–0.433

DMN: default mode network; LFr: left fronto-parietal network; RFr: right fronto-parietal network; mVis: medial visual network; AN: audial network; ECN: executive control network; OccP: occipital pole network; ?: IC does not match any classical network; MADRS: Montgomery-Asberg depression rating scale; BDI: Beck depression inventory; ZSRDS: Zung self-rating depression scale; ^∗^*p* < 0.05; ^∗∗^*p* < 0.01; ^a^pre-post difference in connectivity of this pair is significant for this condition.

**Table 11:** Correlation of IC maps from the patients vs. controls study (left column) and from dynamics study (right column).

IC no.	Best match
1 (DMN)	10, DMN (*r* = 0.85)
2 (RFr)	7, RFr (*r* = 0.78)
3	2 (*r* = 0.69), n/a
4	8 (*r* = 0.81), n/a
5 (mVis)	1, mVis (*r* = 0.85)
6	3, OccP (*r* = 0.39); 9, lVis (*r* = –0.55)
7	13, SMN (*r* = 0.75)
8 (AN)	5, AN (*r* = 0.72)
9 (LFr)	15, LFr (*r* = 0.74)
10 (lVis, OccP)	3, OccP (*r* = 0.58); 9, lVis (*r* = 0.46)
11 (vDMN)	16, DMN (*r* = 0.57); 17 (*r* = 0.56)
12	5, AN (*r* = 0.19)
13 (ECN, dDMN)	11, ECN (*r* = 0.63)
14	4 (*r* = 0.69), n/a
15	6 (*r* = –0.64), n/a
16 (DMN)	12, DMN, LFR (*r* = 0.41); 16, DMN (*r* = 0.41)
17 (LN)	12, DMN, LFR (*r* = 0.55)
18	14 (*r* = 0.57); 11, ECN (*r* = 0.41)
19	17 (*r* = –0.22)
20 (Cer)	2 (*r* = 0.39), n/a

(v)DMN: (ventral) default mode network; LFr: left fronto-parietal network; RFr: right fronto-parietal network; Cer: cerebellar network; mVis: medial visual network; AN: audial network; LN: language network; ECN: executive control network; SMN: sensorimotor network; OccP: occipital pole network; n/a: artifact or does not correspond to any classical network; *r*: correlation coefficient.

## Data Availability

The MRI and fMRI datasets generated for this study can be downloaded from http://openneuro.org/ repository without any access restriction, study 1 https://openneuro.org/datasets/ds002748, study 2 https://openneuro.org/datasets/ds003007.
